
JOURNAL INFORMATION
==============================
NLM Title Abbreviation: Hist Cienc Saude Manguinhos
Journal ID: Hist Cienc Saude Manguinhos
NLM Journal ID: 9513999
Journal ID: hcsm

História, Ciências, Saúde - Manguinhos

ISSN: 0104-5970
EISSN: 1678-4758
Publisher: Casa de Oswaldo Cruz - Fiocruz

ARTICLE INFORMATION
==============================
PMCID: PMC10494972
DOI: 10.1590/S0104-59702023000100043
Article ID: 01502
Article version: 1
Subjects: Resenhas

History and the social sciences respond to the covid-19 pandemic

A história e as ciências sociais respondem à pandemia de covid-19

http://orcid.org/0000-0002-9291-7232 Cueto Marcos i
i Pesquisador, Casa de Oswaldo Cruz/Fiocruz. Rio de Janeiro – RJ – Brasil cuemarcos@gmail.com
Electronic publication date: 2023 Sep 4
Publication date: 2023
Volume: 30
Issue: Suppl 1
Electronic Location ID: e2023043
License: This is an Open Access article distributed under the terms of the Creative Commons Attribution License, which permits unrestricted use, distribution, and reproduction in any medium, provided the original work is properly cited.
License URL: https://creativecommons.org/licenses/by/4.0/

Figure count: 1
Table count: 0
Equation count: 0
Reference count: 3
Page count: NaN

==============================

Com a exacerbação e expansão da pandemia de covid-19 nos primeiros quatro meses de 2020, ficou evidente que não se tratava apenas de uma crise sanitária emergente de origem zoonótica. A presença do SARS-CoV-2 trouxe consigo a necessidade de resgate de estudos sobre as dimensões sociais, econômicas, culturais e políticas das experiências pandêmicas ao longo da história. Nessa direção, mais de 65 autores (entre os quais vários historiadores destacados como Simone Petraglia Kropf, Paloma Porto, Ede Cerqueira, André Luiz da Silva Lima e Carlos Machado de Freitas) tentam, nesse valioso livro, compreender os impactos diferenciados da pandemia sob perspectivas interdisciplinares ancoradas nas ciências humanas e sociais. Disponível em acesso aberto, o livro oferece uma crônica clara e ordenada da pandemia. Ele versa sobre os debates da dicotomia saúde-economia, aponta as primeiras restrições urbanas e as criações de hospitais de campanha, expõe as dificuldades e limitações de testagem e diagnóstico da doença, ressalta a popularidade de posturas negacionistas e anticientíficas em alguns setores, promotoras de penosas adversidades no trabalho do SUS. Muitos dos tenebrosos eventos ocorridos no Brasil pandêmico foram motivados pelas barbaridades do presidente Jair Bolsonaro, em razão da errônea concepção de imunidade de rebanho, fruto de sua irracionalidade desumana, negligência consciente, necropolítica de Estado e banalização da morte dos mais pobres (Caponi, 2021).

Os autores destacam como o eurocentrismo moldou as respostas brasileiras ante a pandemia. Os seguidores do modelo europeu replicaram os isolamentos breves seguidos de reaberturas precoces decretadas pelas autoridades estaduais e municipais independentemente do número de casos locais. Além disso, mais de um capítulo do livro analisa de maneira criativa as vozes das favelas e das populações indígenas numa metodologia de produção compartilhada de conhecimento, com o fito de exibir o papel das populações vulneráveis de bairros periféricos e sua atuação na criação de ações de resistência ao poder centralizado das autoridades sanitárias e à doença em si. Para garantir o exercício dos direitos sociais dos mais pobres, surgiram nesses territórios narrativas e movimentos sociais organizados alternativos ao Estado. Foram mobilizadas redes de solidariedade em favelas e regiões marginalizadas, especializadas em identificar famílias vulnerabilizadas com fins de fornecer alimentos e *kits* anti-covid-19, contendo itens de higiene, máscaras e álcool. Em constante parceria com a Fiocruz, barreiras sanitárias foram instituídas no acesso às aldeias indígenas para controlar a circulação de pessoas estranhas, minimizar os impactos gerados pelas sucessivas quarentenas e a indiferença do governo.

Outrossim, o livro indica as principais iniciativas brasileiras e internacionais orientadas pelas ciências sociais e humanidades para compreender a emergência sanitária. Sem correr grandes riscos, é possível afirmar que uma das mais relevantes criações foi o Observatório Covid-19 BR da Fundação Oswaldo Cruz (Fiocruz), lançado em abril de 2020. Essa iniciativa foi pensada para desenvolver investigações, tecnologias e propostas de divulgação contínua de informações atualizadas com alto rigor científico. Os capítulos finais do livro – parte da terceira e última seção, "Ciência, tecnologia e comunicação" – descrevem a atuação da Fiocruz em suas diversas frentes de enfrentamento à covid-19.

A obra salienta a coordenação da instituição nos ensaios clínicos de vacinas, intitulado Solidariedade, lançado em março de 2020 pela OMS, bem como destaca a inovadora parceria com os idealizadores da vacina AstraZeneca em um histórico acordo de transferência de tecnologia para a Fiocruz. Igualmente, o livro descreve os passos para a produção de *kits* para testes moleculares do novo coronavírus e assinala a participação dos cientistas de Manguinhos na promoção de diversas redes transnacionais de pesquisas e formação continuada de profissionais posicionados na linha de frente de combate à pandemia. Fica visível o protagonismo da Fundação Oswaldo Cruz na articulação de lideranças sociais e políticas heterogêneas, entre outras intervenções para além das intervenções biomédicas, como o fomento de editais de apoio econômico às comunidades periféricas em situações de vulnerabilidade social.

A maioria dos textos parte de quatro pressupostos. Primeiramente, embora o vírus SARS-CoV-2 tenha sido difundido em poucos meses em mais de duzentos países, o seu impacto não foi igual em todas as partes do mundo. Essa pandemia precisa ser compreendida a partir de categorias vinculadas a disparidades etárias, políticas e econômicas e deve ser analisada em face dos marcadores de dominação do capital, iniquidades estruturais como raça, etnicidade, gênero, classe social, e assimetrias econômicas entre os países impactados. Em segundo lugar, são ressaltados os impactos mais graves ocasionados pelo descaso do governo brasileiro ao enfraquecer as políticas de proteção social desde o golpe sofrido pela presidenta Dilma Rousseff em 2016. Em seguida, o livro expõe como precisamos recorrer as reflexões teóricas e práticas das ciências sociais e da saúde pública – como os estudos sociais da ciência e tecnologia e da saúde coletiva brasileira – para compreender e responder de forma célere e democrática à robusta e complexa catástrofe humanitária provocada pela covid-19. E por fim, os textos reunidos nesse trabalho reforçam como a Fiocruz possui experiência notória e conhecimentos acumulados por várias décadas capazes de garantir contribuições biomédicas e subsídios para fundamentar a elaboração de políticas públicas indispensáveis aos brasileiros e à ciência mundial na luta contra pandemias vindouras.

Os organizadores acreditam que os desafios colocados pela pandemia impõem agora e no futuro a necessidade de reimaginar as ciências sociais e humanas numa perspectiva interdisciplinar. Daí a necessidade de chamar a atenção para três contribuições fundamentais, pouco comuns entre os historiadores em nossos tempos, quais sejam: estimular o interesse pela pesquisa de assuntos contemporâneos, participar da construção de redes com profissionais de áreas científicas não relacionadas às ciências humanas, e finalmente, conectar as investigações históricas com o fito de fortalecer ações voltadas para auxiliar o enfrentamento de uma pandemia. Esses caminhos incluem e vão além dos estudos do tempo presente e do conhecido conceito do filósofo francês Bruno Latour, de observar a ciência em ação.

Como em muitas coletâneas, a maioria dos capítulos pode ser lida independentemente. Embora alguns temas, assuntos e instituições se repitam, a bioética e a saúde indígena mereceriam um tratamento mais extenso nos próximos volumes. Assuntos interessantes como o uso oficial da cloroquina, receberam pouca atenção pese a presença do debate sobre o medicamento no excelente capítulo final, "Covid-19 nas mídias: medo e confiança em tempos de pandemia", escrito por Kátia Lerner, Janine Miranda Cardoso e Tatiana Clébicar. Uma pergunta surge como efeito da publicação desse importante texto. Afinal, nos últimos meses pandêmicos, os estudos interdisciplinares e as políticas sociais foram conjugados às respostas biomédicas? A meu ver, isso não ocorreu.

Como os textos foram elaborados entre novembro de 2020 e janeiro de 2021, os autores do livro não podiam antecipar o que aconteceria na segunda metade de 2021. Tudo indica que na retaguarda das vacinas – impulsionadas com força no Brasil desde meados de 2021– se escondiam poderosas decisões neoliberais e biomédicas para as quais as ciências sociais e valores como a integralidade, universalidade e equidade não foram relevantes para moldar as políticas de saúde brasileiras durante a pandemia da covid-19 (Cueto, Neelakantan, Lopes, 2022). Isso ficou claro em dois casos. Primeiro, o tratamento foi considerado uma mercadoria e não um bem público. Por exemplo, desde os trágicos últimos meses de 2020 e durante 2021, os países industrializados – com apoio do governo de Bolsonaro – se opuseram aos pedidos da Índia e da África do Sul de suspender no decorrer da pandemia as patentes dos imunizantes e equipamentos médicos. Em segundo lugar, os países ricos recriaram as fronteiras entre o norte global e o sul global (por exemplo, açambarcavam vacinas e na prática boicotavam a iniciativa multilateral Covax Facility de fornecer imunizantes aos países em desenvolvimento).

Suspeito que, ao menos desde a segunda metade de 2021, a biomedicalização foi hegemônica na maioria das respostas políticas à covid-19. Ao mesmo tempo os chamados iniciais à equidade social para enfrentar a pandemia e aliviar os problemas de saúde perderam forças. Em todo caso esse livro lembra que apesar das visões abrangentes, interdisciplinares e críticas fornecidas pela história da saúde e ciências sociais, a luta pelo reconhecimento desses campos como importantes aliados no combate às pandemias do porvir continua.


REFERÊNCIAS

CAPONI Sandra Covid-19 em Santa Catarina: um triste experimento populacional História, Ciências, Saúde – Manguinhos 28 2 593 598 2021 34190797 10.1590/S0104-59702021005000004
CUETO Marcos NEELAKANTAN Vivek LOPES Gabriel The regulation of necropolitics: governmental responses to covid-19 in Brazil and India in the first year of the pandemic SciELO Preprints 10.1590/SciELOPreprints.4244 2020 Acesso em: 6 fev. 2023
MATTA Gustavo Corrêa et al org Os impactos sociais da covid-19 no Brasil: populações vulnerabilizadas e respostas à pandemia Rio de Janeiro Editora Fiocruz 2021 E-book https://books.scielo.org/id/r3hc2 Acesso em: 7 fev. 2023
